# Novel Antiplasmodial Natural Products Identified Through a Modified Bioluminescence-Based Rate-of-Kill Assay

**DOI:** 10.3390/biomedicines14030585

**Published:** 2026-03-05

**Authors:** Rebecca Mobley, Suzanne A. Nasser, Barbara Bartholomew, Robert Nash, Paul Horrocks, Helen Price

**Affiliations:** 1 School of Medicine, Keele University, Keele, Staffordshire ST5 5BG, UK; 2 PhytoQuest Limited, Aberystwyth SY23 3EB, UK; 3 School of Life Sciences, Keele University, Keele, Staffordshire ST5 5BG, UK; h.price@keele.ac.uk

**Keywords:** antiplasmodial activity, natural product library, *plasmodium falciparum*, modified bioluminescence-based rate of kill, intraerythrocytic activity

## Abstract

**Background/Objectives**: The discovery of antimalarial compounds with novel mechanisms of action and distinct rates of kill (RoK) is essential to address emerging drug resistance in *Plasmodium falciparum*. Natural product libraries represent a valuable and chemically diverse source of potential new antiplasmodial scaffolds. This study aimed (i) to evaluate a modified bioluminescence relative rate-of-kill (mBRRoK) assay as a rapid triage platform for screening large compound libraries with previously unknown antiplasmodial activity, enabling simultaneous assessment of potency and RoK, and (ii) to identify novel compounds with potent and selective in vitro erythrocytic activity. **Methods:** A fixed two-concentration mBRRoK screen was applied to 1165 compounds from the PhytoQuest natural product library. Antiplasmodial activity and RoK profiles were assessed over 48 h using two genetically distinct luciferase-expressing *P. falciparum* strains (Dd2^luc^ and NF54^luc^) with distinct drug resistance backgrounds. Reproducibility was evaluated across biological replicates. Selected hits underwent secondary profiling, including EC_50_ determination and HepG2 cytotoxicity assessment to establish potency and selectivity. **Results**: The primary screen identified 36 lead compounds demonstrating potent activity within 48 h, encompassing both fast- and slow-acting phenotypes. Activity was reproducible and largely strain-independent across both parasite lines. Secondary profiling prioritised four compounds (**100657**, **101158**, **101160**, and **101173**) with nanomolar-to-micromolar antiplasmodial potency and favourable selectivity indices relative to mammalian cell cytotoxicity. **Conclusions**: The mBRRoK assay provides a robust and scalable framework for integrating potency and pharmacodynamic assessment in early antimalarial discovery. This strategy enabled efficient prioritisation of selective natural product-derived leads with distinct killing profiles, supporting their progression toward further optimisation and preclinical development.

## 1. Introduction

The emergence of resistance to frontline artemisinin combination therapy underscores the urgent need for new antimalarial drugs with novel mechanisms of action [1]. The limited structural diversity of current antimalarials further increases the risk of resistance development. Natural products offer a rich source of chemically diverse compounds with potential as novel therapeutics or chemical probes [2,3]. Many existing antimalarials, including artemisinin derivatives and quinine-related compounds, are derived from natural sources. Despite this, microbial natural product libraries remain relatively underexplored for antiplasmodial activity [1,4].

In this study, a library of 1165 natural products provided by PhytoQuest was screened for antiplasmodial activity. The chemical diversity of the library spans several major classes, including carbohydrates, macrocyclic lactones and lactams, quinones, amino acid derivatives and peptides, nitrogen- or sulfur-containing heterocycles, as well as alicyclic, aromatic, and aliphatic compounds. Classification of compounds into these broad structural groups followed the Bérdy [5] system, which categorises natural products based on biosynthetic origin and chemical scaffold. The library is mostly sourced from fungi, but also includes compounds isolated from actinomycetes and plants. These compounds were isolated from solid flask cultures grown under conditions that promote secondary metabolite diversity. Compound selection prioritised drug-like properties, including molecular weight and lipophilicity within Lipinski’s parameters. Previous screening of a PhytoQuest plant-derived library identified potent antiparasitic candidates against multiple protozoa, but none with adequate selectivity against *P. falciparum* [6]. The present study aimed to evaluate a novel natural product library for potential antiplasmodial activity.

Rapid high-throughput screening is a central component of modern antimalarial drug discovery. Obtaining information on key compound properties early in the drug discovery process is essential for prioritising promising candidates and minimising downstream attrition [7]. Among these tools, the bioluminescence relative-rate-of-kill (BRRoK) assay offers a rapid, semi-quantitative measure of initial cytocidal activity using luciferase-expressing *P. falciparum* [8,9]. The BRRoK assay captures the early cytocidal effect of a compound within 6 h, ranked against benchmark controls with well-defined RoK profiles [8,9].

However, the traditional BRRoK assay requires pre-determined EC_50_ values for each compound, limiting its application to libraries of compounds with unknown activity. To address this limitation, the modified BRRoK (mBRRoK) assay was developed [8], utilising two fixed concentrations against synchronised trophozoites for 6 h, to rapidly triage large libraries and identify potent and rapid-acting candidates without prior EC_50_ determination. In this assay, bioluminescence loss is proportional to both the cytocidal rate and potency of the compound, supporting efficient prioritisation of leads for further evaluation [8].

The robustness of the mBRRoK assay was demonstrated through screening of the TCAMS library (~12,000 compounds) of known antiplasmodial activity, which identified 975 compounds with cytocidal profiles comparable to or exceeding that of chloroquine. This dual concentration design allowed for rapid triaging of large compound sets while maintaining the ability to distinguish potent, fast-acting compounds from slower-acting candidates during preliminary screening [8]. These results highlighted the assay’s strong predictive value and its capacity to streamline the identification of candidates within large diverse chemical libraries.

Building on these advances, the current study had two primary objectives: first, to adapt and validate the mBRRoK assay for natural product libraries of unknow antiplasmodial activity; and second, to identify novel antiplasmodial candidates from the PhytoQuest microbial natural product library [10,11].

## 2. Materials and Methods

### 2.1. Source and Composition of the PhytoQuest Natural Product Library

A new library of 1165 natural products was provided by PhytoQuest Ltd. (Aberystwyth, UK) for antiparasitic screening. The library was designed to maximise chemical and biological diversity and consists of compounds isolated and purified from solid microbial flask cultures, rather than the more commonly used shake-flask mycelial cultures. This solid-phase cultivation method promotes secondary metabolite production, resulting in a unique library enriched in structurally diverse and bioactive compounds (PhytoQuest).

The library was supplied in mass-per-volume format (1 mg/mL in DMSO) and was stored at −20 °C. The compounds had a broad range of molecular weights (mean MW ≈ 340 g/mol). Based on prior mBRRoK optimisation [8] and the anticipated lower potency of natural products compared with synthetic reference libraries, two screening concentrations were selected to ensure broad detection sensitivity. These corresponded to approximately 20 μM (“high”) and 4 μM (“low”), equivalent to final working concentrations of 6.8 μg/mL and 1.44 μg/mL, respectively.

### 2.2. Plasmodium Falciparum Culture

All *P. falciparum* culture work was conducted in a Category III containment facility in accordance with a Health and Safety Executive-approved code of practice. Parasites were maintained using a method adapted from Trager and Jensen [12]. Cultures were maintained at 2% haematocrit (HCT) with parasitaemia typically in the range of 0.5–5%. Two transgenic *P. falciparum* strains were used for in vitro compound screening: the multi-drug-resistant Dd2^luc^ and the drug-sensitive NF54^luc^. Both reporter lines were genetically engineered to express luciferase and were developed as previously described [13,14]. The Dd2^luc^ strain expresses luciferase at the trophozoite stage under proliferating cell nuclear antigen (PCNA) regulatory sequences and uses blasticidin S deaminase for selection [13]. The genetically distinct NF54^luc^ was later developed to express luciferase using the same reporter and regulatory elements and the human dihydrofolate reductase for selection with WR99210 [14].

### 2.3. HepG2 Cell Culture

HepG2 cells, a human hepatocellular carcinoma–derived epithelial cell line, were obtained from the European Collection of Authenticated Cell Cultures (ECACC) (Sigma-Aldrich, St. Louis, MI, USA). Cells were maintained in Dulbecco’s Modified Eagle Medium (DMEM; Sigma) supplemented with 10% foetal bovine serum (FBS; Gibco, Grand Island, NY, USA) and 2.5 μg/mL penicillin–streptomycin, (Sigma) and incubated at 37 °C in a humidified atmosphere containing 5% CO_2_. Cultures were routinely monitored for confluency and sub-cultured at 80–90% confluency using trypsinisation. Medium was replaced twice weekly.

#### HepG2 Cytotoxicity and Viability Assays

Cells were harvested at ~80% confluency, detached with 1× trypsin (Gibco), and counted using a hemocytometer. Cells were seeded at 1 × 10^5^ cells/mL, with 200 μL per well in 96-well plates. For cytotoxicity screening, compounds were added in duplicate across three independent biological repeats (*n* = 6) at 20 μM and mixed by pipetting. For cell viability growth curves, 2-fold serial dilutions of compounds were performed in duplicate. Plates included untreated controls and 1 μM actinomycin D as a positive control. Plates were incubated at 37 °C with 5% CO_2_ for 48 h.

Cell viability was assessed using the PrestoBlue assay. Twenty microliters of PrestoBlue reagent (1:10 in medium) (Thermo Fisher Scientific, Waltham, MA, USA) was added per well and incubated for 2 hours at 37 °C, 5% CO_2_. Fluorescence was measured using a Glomax Multi Detection System (Promega, UK; excitation 525 nm, emission 650 nm).

### 2.4. Bioluminescence Relative Rate-of-Kill (BRRoK) Assay

The BRRoK assay was used to determine the dose- and time-dependent inhibition of *Plasmodium falciparum* and to characterise compound RoK relative to benchmark antimalarials, as previously described [9] with minor modifications. Early trophozoite-stage parasites (~18–24 hours post-infection, confirmed by microscopy) were prepared at 1–2% parasitaemia and 2% HCT as a master mix.

For initial screening, the full PhytoQuest microbial library (1165 compounds) was tested at two fixed concentrations (6.8 µg/mL [high] and 1.36 µg/mL [low]) in 96-well plates, with 24 compounds per plate tested in triplicate. Chloroquine (Sigma-Aldrich), mefloquine (Sigma-Aldrich), and atovaquone (USP, Washington, DC, USA) were included as benchmark controls at high (20 μM) and low (4 μM) concentrations, alongside an untreated positive control. Plates were incubated at 37 °C in a sealed gas chamber (1% O_2_, 3% CO_2_, 96% N_2_), and bioluminescence was measured at 6 and 48 hours using the luciferase assay. These time points were selected to capture both rapid-acting compounds targeting trophozoite stages (6 h) and slower-acting compounds exerting effects across the full erythrocytic cycle (48 h), consistent with combination therapy principles that combine fast- and slow-acting drugs to enhance efficacy and treatment outcomes [10,11]. Growth was expressed relative to the untreated control. The full library (1165 compounds) was screened once (*n* = 1) against Dd2^luc^, and the 36 most potent compounds were re-tested in triplicate (*n* = 3) against both Dd2^luc^ and NF54^luc^ lines.

Compounds were also assessed in BRRoK assays using three-fold dilutions starting at 9× EC_50_, with 1–2% synchronised early trophozoites, incubated for 48 h. Bioluminescence was measured at 6 hours and 48 hours, and % normalised growth was plotted against ×EC_50_. Chloroquine and atovaquone were included as reference controls for fast- and slow-acting activity, respectively.

### 2.5. Luciferase Bioluminescence Assay

Luciferase bioluminescence assays were used to quantify *P. falciparum* growth and viability, following the method of Hasenkamp et al. [15] with minor modifications. After 48 h of incubation with test compounds, 40 μL of culture was transferred to a white 96-well assay plate containing 10 μL of 5× Passive Lysis Buffer (Promega, Madison, WI, USA). The contents were mixed gently, and 50 μL of Luciferase Assay Reagent (Promega) was added immediately before reading. Luminescence was measured using a Glomax Multi Detection System (Promega) with a 0.5 s integration time.

The bioluminescence signal, expressed as relative light units (RLU), was recorded and used as a direct measure of parasite viability. Bioluminescence values were normalised against untreated parasite controls to determine percentage growth inhibition. A supralethal dose of chloroquine (10 μM) was included as a negative control on each plate. All assays were performed in triplicate, and data were analysed as the mean percentage of normalised bioluminescence relative to untreated controls.

### 2.6. Data Analysis

All data were analysed using GraphPad Prism (version 9.4.1, GraphPad Software, San Diego, CA, USA). Dose–response curves were generated by nonlinear regression using a four-parameter logistic model to estimate EC_50_ values and corresponding 95% confidence intervals. Where applicable, data are presented as mean ± SD from at least two independent biological replicates.

## 3. Results

### 3.1. Fixed-Concentration Screening of the PhytoQuest Natural Product Library

**Figure 1:**
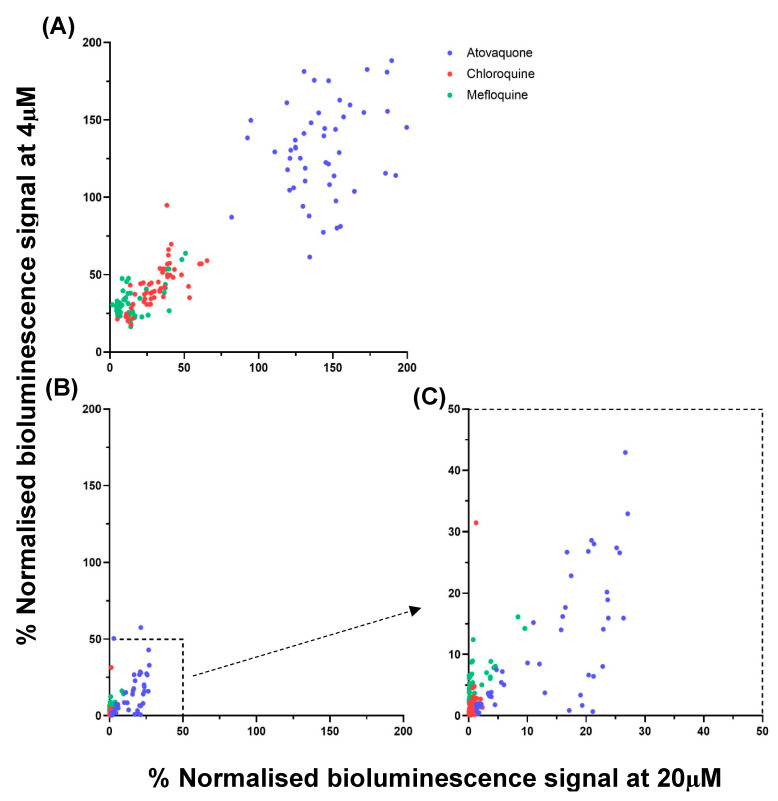
Scatter plots of % normalised bioluminescence signal of *P. falciparum* Dd2^luc^ following incubation with rapid and slow antimalarial controls. Controls: chloroquine (red), mefloquine (green), and atovaquone (blue) were incubated at [high] (20 μM) and [low] (4 μM) and the % bioluminescence signal remaining, compared to matched untreated control on the same plate plotted at 6 h (**A**) and 48 h (**B**). Each data point represents *n* = 1 biological repeat. (**C**) shows data points for the 48-h incubation with axis cropped and expanded to better show the lower left quadrant of (**B**).

Overall, benchmark antimalarials—chloroquine, mefloquine, and atovaquone—showed consistent, reproducible, and expected activity across 52 screening plates. Chloroquine and mefloquine showed rapid growth inhibition, with normalised bioluminescence dropping to <5% by 48 h, while atovaquone exhibited delayed activity, consistent with its slow-acting profile (Figure 1).

**Figure 2:**
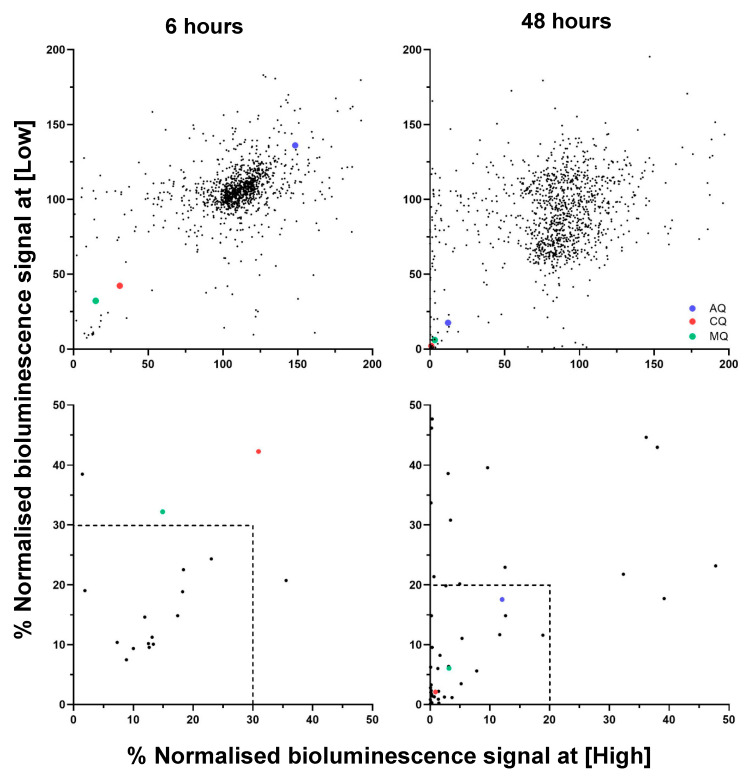
mBRRoK plots of the PhytoQuest natural product library. Synchronised Dd2^luc^ cultures (1–2% parasitaemia, 2% HCT) were incubated with a high concentration (6.8 μg/mL) and low concentration (1.4 μg/mL) of test compound for 48 h (*n* = 1). The % normalised bioluminescence signal was established at 6 h (graphs on the left) and 48 h (graphs on the right). mBRRoK plots on the **top row** show data points for all compounds from the PhytoQuest library and those on the **bottom row** show data points with cropped and expanded axis from the graph shown immediately above. Lead compound criteria of <30% signal for 6 h data and <20% signal for 48 h data are represented by the dashed-line boxes. The mean % normalised bioluminescence signal of controls CQ—chloroquine (red) AQ—atovaquone (blue), and MQ—mefloquine (green) (*n* = 52) are shown as key landmarks on these mBRRoK plots.

Screening of the 1165 PhytoQuest natural products against synchronised *P. falciparum* Dd2^luc^ early trophozoites at two fixed concentrations (6.8 µg/mL and 1.4 µg/mL) showed that most compounds clustered in the top right quadrant of the mBRRoK plots at both 6 h and 48 h, indicating limited or no antiplasmodial activity. A subset of compounds appeared in the bottom left quadrant, particularly at 48 h, indicating growth inhibition and identifying potential lead candidates (Figure 2).

Using benchmark-based thresholds, 13 compounds demonstrating ≤ 30% normalised bioluminescence at 6 h exposure were classified as putative fast-acting hits. An additional 23 compounds with <20% bioluminescence at 48 h were identified as putative slow-acting hits, yielding 36 preliminary leads (Supplementary Figure S1).

**Figure 3:**
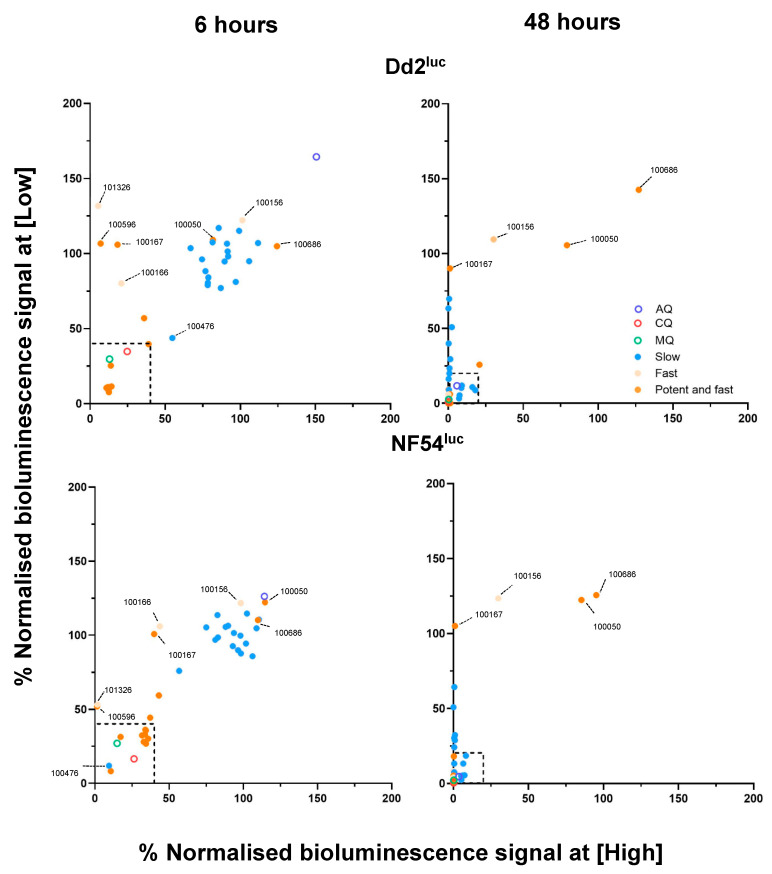
mBRRoK screen of lead microbial products against *P. falciparum* Dd2^luc^ and NF ^luc^. Microbial natural products with potential potent and fast (orange), less potent and fast (beige), and slow (blue) antiplasmodial activity screened against *P. falciparum* Dd2^luc^ (**top row**) and NF54^luc^ (**bottom row**). Compounds were incubated with synchronised 1–2% trophozoites (2%HCT) for 48 h. The % normalised bioluminescence signal at 6 h and 48 h (*n* = 3, mean plotted). CQ—chloroquine (red ring), MQ—mefloquine (green ring), and AQ—atovaquone (blue ring) (*n* = 6). Black dashed-line squares mark criteria for lead compounds from the previous fixed two-concentration screen.

Re-screening of the 36 lead PhytoQuest compounds against Dd2^luc^ in three biological replicates showed that 27 compounds retained consistent activity at 6 h, and 25 compounds maintained antiplasmodial activity at 48 h (Figure 3). Several compounds exhibited reduced potency, particularly at lower concentrations, while two compounds (**100050** and **100686**) showed no reproducible activity and were excluded. Overall, the majority of fast-acting and slow-acting classifications were maintained, indicating general reproducibility of the fixed-concentration screen, with variability primarily reflecting differences in potency rather than RoK.

### 3.2. Activity of Lead Compounds Against P. Falciparum NF54^luc^

The 36 leads were also screened against the genetically distinct NF54^luc^ strain. Most compounds occupied similar positions on mBRRoK plots for both strains, indicating largely strain-independent activity (Figure 3).

Interestingly, a greater reduction in normalised bioluminescence at both 6 h and 48 h was observed with compound **100476** in NF54^luc^ compared with Dd2^luc^. This differential activity was reproducible across biological replicates, indicating increased potency of compound **100476** in the chloroquine-sensitive NF54 genetic background. Compounds **100050** and **100686** showed no measurable inhibition in both Dd2^luc^ and NF54^luc^ assays and were excluded from further analysis.

Compounds **100166**, **100167**, **100596**, and **101326** showed growth inhibition at 6 h only at the higher concentration, occupying the top-left quadrant of the mBRRoK plots indicative of rapid but less potent activity, against both Dd2^luc^ and NF54^luc^ (Figure 3). Compound **100156**, initially classified as fast-acting with reduced potency, showed variable inhibition between screens, demonstrating minimal activity at 6 h and partial inhibition at 48 h only at the higher concentration (Figure 3). Together, these data indicate that while most lead compounds retained consistent RoK classifications across strains, several exhibited reduced or strain-dependent potency at lower concentrations.

### 3.3. Concentration-Dependent Growth Inhibition

To prioritise compounds for EC_50_ determination, 34 compounds were tested against Dd2^luc^ and 33 compounds against NF54^luc^ at 3, 1, and 0.3 µg/mL. All compounds exhibited concentration-dependent inhibition at 48 h consistent with activity demonstrated in the mBRRoK assay (Supplementary Figure S2). Eleven compounds (**100180**, **100181**, **100585**, **100648**, **100657**, **100669**, **100670**, **100705**, **100735**, **101158**, and **101160**) reduced Dd2^luc^ parasite growth to <2% at all three concentrations tested. Ten of these compounds, excluding **100735**, showed comparable activity against NF54^luc^ and were prioritised for EC_50_ determination.

### 3.4. EC_50_ Determination

Based on the three-concentration screening data, nine compounds (**100180**, **100181**, **100585**, **100648**, **100657**, **100669**, **100705**, **101158**, and **101160**) with comparable activity to chloroquine, mefloquine, and atovaquone (<2% growth at all three concentrations) were selected for EC_50_ determination against *P. falciparum* Dd2^luc^. A consistent EC_50_ could not be determined for compound **100670** despite repeated attempts, and was excluded from further analysis. Compound **100570**, which narrowly missed the original selection criteria but retained appreciable activity (NF54^luc^ < 10% growth; Dd2^luc^ 28% growth at 0.3 µg/mL), was included as a replacement. In addition, compound **101173** was included due to its relatively potent and potentially selective antiplasmodial activity observed in earlier screens. To assess strain-independent activity, EC_50_ determination was extended to NF54^luc^ for six of these compounds, comprising three predicted fast-acting and three predicted slow-acting candidates.

Across both strains, the majority of compounds exhibited EC_50_ values ranging from 1.1 µg/mL to 1.5 ng/mL, corresponding to ~2 µM to 2.8 nM when adjusted for molecular weight. The most potent compounds were **100585** (Dd2^luc^: 3.2 ng/mL; NF54^luc^: 1.5 ng/mL), **100669** (12.8 ng/mL, Dd2^luc^), **100181** (Dd2^luc^: 30.6 ng/mL; NF54^luc^: 23.9 ng/mL), and **101158** (Dd2^luc^: 48 ng/mL; NF54^luc^: 72.5 ng/mL), which demonstrated potency comparable to benchmark antimalarials. EC_50_ values were generally comparable between Dd2^luc^ and NF54^luc^, indicating largely strain-independent antiplasmodial potency (Table 1).

### 3.5. Rate-of-Kill Validation Using BRRoK

Growth inhibition assays conducted throughout this study indicated that lead compounds from the PhytoQuest natural product library exhibited mostly comparable antiplasmodial activity against both *P. falciparum* Dd2^luc^ and NF54^luc^, with consistent predicted rates of kill derived from the mBRRoK analysis. Full BRRoK profiling confirmed these predictions. Among the fast-acting compounds, **100181**, **100180**, **100585**, **100648**, and **100657** produced rapid growth inhibition within 6 h, comparable to chloroquine. The proposed slow-acting compounds—**100570**, **101158**, and **101160**—displayed delayed antiplasmodial activity, with minimal inhibition at 6 h and marked growth suppression only at 48 h, similar to atovaquone (Supplementary Figure S3).

Direct comparison of BRRoK profiles for four representative compounds (**100181**, **100648**, **101158**, and **101160**) demonstrated consistent RoK behaviour between Dd2^luc^ and NF54^luc^ parasites (Supplementary Figure S4). Together, these data validate the fixed two-concentration screening approach as an effective predictor of parasite RoK across genetically distinct *P. falciparum* strains.

### 3.6. Cytotoxicity and Selectivity in HepG2 Cells

Six compounds (**100181**, **100570**, **100657**, **101158**, **110160**, and **101173**) were assessed for cytotoxicity against HepG2 cells. Compounds **100657**, **101158**, **101160**, and **101173** showed no detectable cytotoxicity at 20 µM and subsequent cytotoxicity testing established high minimum selectivity indices of >8 to >211 nM (Table 2). Compounds **100181** and **100570** reduced HepG2 viability at 20 µM and EC_50_ determination indicated selectivity indices of 102–130 and 41, respectively (Table 2).

Overall, four compounds—**100657**, **101158**, **101160**, and **101173**—were identified as strong candidates for further preclinical evaluation. These molecules demonstrated potent antiplasmodial activity, low mammalian cytotoxicity, and defined fast-(**100657**) and slow-acting (**101158**, **101160** and **101173**) RoK profiles against *P. falciparum* Dd2^luc^ and NF54^luc^ in vitro.

## 4. Discussion

The discovery of novel antimalarial compounds with distinct mechanisms of action and RoK remains a critical priority in the context of increasing *P. falciparum* resistance to frontline therapies [1]. In this study, application of the mBRRoK assay was extended beyond its original validation [8] to screen a chemically diverse microbial natural product library of unknown antiplasmodial activity. Findings showed that the mBRRoK framework triaged compound potency and RoK simultaneously within a single, scalable assay, providing a powerful early-stage prioritisation tool for antimalarial drug discovery.

Application of the mBRRoK assay to the PhytoQuest library (1165 compounds) identified 36 compounds with reproducible antiplasmodial activity in an initial fixed-concentration screen against *P. falciparum* Dd2^luc^. This screen captured both fast- and slow-acting profiles, extending the utility of the assay beyond rapidly cytocidal compounds and supports the broader goal of identifying combination therapies that pair rapid clearance with sustained suppression [10,11]. This study showed that while a 6 h mBRRoK readout is sufficient for prioritising fast-acting compounds in activity-enriched libraries [8], a 48 h readout is essential for naïve or chemically diverse libraries.

The 48 h mBRRoK plots revealed systematic shifts in compound positioning that were consistent with known antiplasmodial pharmacodynamics. Fast-acting compounds progressed deeper into regions of maximal inhibition, whereas slow-acting compounds occupied higher, overlapping positions, reflecting differences in lag phase and the incomplete attainment of maximal RoK within the 48 h window. Such activity is characteristic of compounds with delayed or indirect mechanisms of action, where parasite killing requires prolonged exposure [16]. Consistent with this, Ullah et al. [11] showed that compounds with short lag phases, such as pyrimethamine (~24 h), produced 48 h BRRoK profiles comparable to the fast-acting drug chloroquine, whereas atovaquone which requires prolonged exposure, exhibited delayed killing. Although atovaquone typically needs more than 48 h to reach maximal parasiticidal activity, the data indicate that a 48 h mBRRoK endpoint is sufficient to identify slow-acting candidates. This approach enables effective discrimination of pharmacodynamic classes without the need for extended incubations that would otherwise reduce assay throughput.

The fixed-concentration design of the mBRRoK assay inherently integrates potency selection alongside RoK assessment. More potent compounds are delivered at higher multiples of their EC_50_, resulting in greater observed inhibition, while less potent compounds may fail to reach the 9/10 × EC_50_ exposure required for maximal RoK [9,16]. Re-screening of prioritised compounds confirmed the overall reproducibility of both potency and RoK predictions in the fixed-concentration mBRRoK assay, with most candidates retaining consistent antiplasmodial activity across biological replicates and genetically distinct parasite strains (Dd2^luc^ and NF54^luc^). These findings support the utility of the mBRRoK assay as a reliable tool for triaging large compound libraries by simultaneously integrating potency and pharmacodynamic behaviour.

Ten compounds demonstrated antiplasmodial activity equivalent to benchmark controls across all tested concentrations and were prioritised for EC_50_ determination. EC_50_ values ranged from low nanomolar to low micromolar concentrations, aligning with predicted potency thresholds established in the mBRRoK assay. All 12 compounds selected for EC_50_ determination against Dd2^luc^ fell within the anticipated range (400 nM–2 μM), with 11 compounds exhibiting EC_50_ values ≤ 657 nM. Similar trends were observed against NF54^luc^, supporting the predictive utility of the mBRRoK framework. These findings demonstrate that appropriate selection of fixed concentrations, guided by the relationship between EC_50_ and RoK, can effectively enrich for compounds with desirable potency profiles.

The use of fixed mass-per-volume concentrations, necessitated by the provision of the PhytoQuest library, imposed additional selectivity based on molecular weight. Higher-molecular-weight compounds were delivered at lower molar concentrations, resulting in more stringent selection. While this may bias against larger molecules, such compounds are often less favourable for oral bioavailability and intracellular penetration [17], suggesting that this bias may be advantageous for triaging libraries early in the drug discovery stage.

Assessment of cytotoxicity is a critical component of early antimalarial drug discovery, as many potent antiplasmodial compounds, particularly natural products, are limited by off-target toxicity [18,19]. In this study, four compounds with activity against *P. falciparum* showed minimal toxicity toward HepG2 cells, indicating a degree of parasite selectivity. This suggests that some compounds with broad antiparasitic activity may offer exploitable therapeutic windows and could serve as promising starting points for medicinal chemistry optimisation aimed at enhancing selectivity for parasite targets over host cells.

Information on the chemical properties and available structures for the 36 lead compounds identified in this study were provided by PhytoQuest, with details presented in the Appendix A. Structural matches were obtained for 16 compounds, revealing enrichment for known classes of bioactive microbial natural products. The most prevalent chemical families included macrocyclic trichothecenes (**100585**, **100670**, **100181**, and **100705**), pyridines (**100288**), cytochalasins (**101159** and **101161**), cladosporins (**100657**), and bis-naphtho-γ-pyrones (**100569**, **100568**, and **100576**). Natural products from these classes have been extensively studied and are well recognised for their diverse biological activities, including antifungal [20], antibacterial [18,19,21], anticancer [22], and antiparasitic effects [22,23].

While fungal metabolites have multiple biological applications, this also lends to a general toxicity of these compounds. This is also apparent for the known natural product structures elucidated in this study. For example, compounds **100585**, **100670**, **100181**, and **100705**, which exhibited potent antiplasmodial activity in this study, were identified as trichothecenes; a class of compounds shown to exhibit antiplasmodial activity [22]. However, this class of compounds have been shown to have general toxicity towards other cell lines, including human cell lines (KB cells, BC1 cells, and Vero cells) [24]. This potentially highlights a limitation for microbial metabolites moving forward. However, Isaka et al. [24] highlighted that while Roridin A and E (trichothecene mycotoxins) show general toxicity toward cell lines (mammalian and parasitic), the activity against *P. falciparum* was more potent than the cytotoxicity toward any other cell line (Isaka et al., 1999). Similarly in this study, compound **100181** (trichoverritone) showed cytotoxicity to HepG2 cells but with a higher selectivity towards *P. falciparum* (selectivity index: Dd2^luc^ 102.0 and NF54 130.5), highlighting the potential for the identification or development of derivative compounds from this library with optimal antiplasmodial action and reduced cytotoxicity.

Exploring the chemical structures revealed consistent mBBRoK profiles for structurally related compounds in this library. This aligns with previous observations that the mBRRoK plots capture functional relatedness in terms of antiplasmodial mode of action, with compounds sharing core scaffolds clustering together [8,9]. Although the antiplasmodial activity of these compounds have not been previously characterised, the activity described here suggests a shared mechanism of action across all compounds sharing structural classes. This finding further extends the utility of the assay in early-stage compound screening.

Despite demonstrating clear utility for triaging large compound libraries with unknown antiplasmodial activity, the mBRRoK approach has several inherent limitations. Firstly, as the fixed two-concentration design intrinsically couples potency and RoK, compounds with lower intrinsic potency may not reach the ≥9–10 × EC_50_ exposure required for maximal RoK discrimination. Consequently, moderately potent fast-acting compounds may be misclassified or overlooked during primary screening.

Secondly, compounds with intermediate or moderate killing kinetics are harder to distinguish on mBRRoK plots, as they often overlap with less potent fast-acting compounds. This ambiguity, previously noted for both BRRoK and mBRRoK formats, limits fine pharmacodynamic resolution without subsequent EC_50_-normalised BRRoK confirmation [8]. For rapid triage of large libraries, however, this limitation is not critical. The stringent bioluminescence thresholds used here (30 × 30% and 20 × 20%) effectively removed weakly active compounds unlikely to be suitable for further development. Although longer exposures can fully characterise delayed-death phenotypes, such approaches reduce throughput and are less suitable for large-scale screening. Capturing informative RoK data within a single erythrocytic cycle therefore offers a practical balance between mechanistic resolution and screening efficiency [9,11].

Thirdly, antiplasmodial activity was assessed using luciferase-expressing Dd2 and NF54 parasite lines, which, while genetically distinct, do not represent the full range of clinically relevant resistance phenotypes. Cytotoxicity testing was also limited to HepG2 cells and may not fully capture broader toxicity risks across diverse mammalian cell types. Moreover, HepG2-based assays may reflect not only the intrinsic effects of parent com-pounds but also potential metabolite-associated liabilities, which could influence appar-ent safety profiles. Differences in media composition and serum content between parasite and mammalian assays may further affect compound availability through protein bind-ing, thereby influencing calculated selectivity indices. Consequently, these values should be interpreted with appropriate caution. Future studies will expand cytotoxicity profiling across additional mammalian cell lines will provide a more comprehensive evaluation of compound safety and pharmacological relevance. In addition, complementary in silico target prediction and broader benchmarking against established antimalarial reference compounds will help elucidate potential mechanisms of action and strengthen translational relevance.

Finally, screening additional *P. falciparum* strains, including artemisinin-resistant lines, as well as evaluating compound activity across other parasite stages such as ring-stage and gametocytes, and development of additional luciferase reporter lines via CRISPR/Cas9 [25,26] could further strengthen the findings of strain- and stage-independent antiplasmodial activity. Collectively, these limitations do not diminish the value of the mBRRoK assay as a high-throughput triage tool but highlight the need for selective follow-up studies incorporating EC_50_-normalised kinetics, expanded parasite panels, and broader toxicity profiling these lead compounds.

Overall, integrating cytotoxicity profiling with mBRRoK-derived pharmacodynamic data allowed prioritisation of compounds that combine potency, RoK characteristics, and selectivity, identifying promising candidates for further chemical refinement. Structure elucidation and scaffold prioritisation of the identified microbial natural products should be pursued, followed by profiling against additional human cell lines and in vitro ADME (absorption, distribution, metabolism, excretion) assays to enhance selectivity and minimise host-cell toxicity. Together, these findings reinforce the value of pairing phenotypic screening with early safety assessment and chemical annotation to guide the rational advancement of natural-product-derived antimalarial candidates.

## 5. Conclusions

The mBRRoK assay provides a robust and scalable platform for screening large libraries of unknown antiplasmodial activity, balancing throughput with pharmacodynamic insight through a 48 h readout and stringent selection criteria. Future work should focus on refining and extending the mBRRoK framework to enhance predictive power and translational relevance in larger, chemically diverse libraries. Overall, these findings validate the mBRRoK assay as a robust tool for efficiently triaging large libraries and highlight several promising compounds suitable for further optimisation and development.

## Figures and Tables

**Table 1:** EC_50_ values of lead compounds from microbial natural product library against *P. falciparum* Dd2^luc^ and NF54^luc^ presented in molar concentration.

		EC_50_ Dd2^luc^			EC_50_ NF54^luc^		
Compound	MW	ng/mL	nM	95% CI	g/mL	nM	95% CI
**100180**	530	95.5	180.0	NR	-	-	-
**100181**	642	30.6	47.6	19.8–47.0	23.9	37.2	15.3–37.3
**100570**	544	162.9	299.4	161.7–164.2	-	-	-
**100585**	530	3.2	6.0	2.9–3.5	1.5	2.8	1.4–1.6
**100648**	359	83.8	233.3	NR	146.7	408.4	126.7–169.8
**100657**	292	169.5	580.2	151.0–190.4	-	-	-
**100669**	614	12.8	20.8	9.3–17.5	-	-	-
**100705**	292	192.3	657.2	168.3–219.7	-	-	-
**101158**	507	48.0	94.65	35.7–64.6	7.25	143.0	58.2–90.4
**101160**	508	92.5	182.0	NR	91.4	179.9	81.1–103.0
**101173**	466	1146	2459	875.6–1500	678.5	1456	493.8–932.4

nM—nanomolar, 95% CI—95% confidence intervals, NR—not reported, (-)—not determined.

**Table 2:** Selectivity of lead microbial natural products for *P. falciparum* cell lines and HepG2 human cell lines. SI = HepG2 EC_50_/*Plasmodium* EC_50_. Where no cytotoxicity was established against HepG2, compound treatment of HepG2 cell at 20μM was used to determine a minimum (>) selectivity index (SI).

	Dd2^luc^ *Plasmodium*		NF54^luc^ *Plasmodium*	
Compound	EC_50_ (nM)	SI	EC_50_ (nM)	SI
**100181**	47.6	102.0	37.2	130.5
**100570**	299.4	41.3	-	-
**100657**	580.2	>34.4	-	-
**101158**	94.65	>211.3	143.0	>139.9
**101160**	182.0	>109.8	179.9	>111.2
**101173**	2459	>8.1	1456	>13.7

## Data Availability

The natural product library used in this study is proprietary and available exclusively from PhytoQuest. Compound chemical formulas and molecular weights for the 36 lead natural products are provided in the Appendix A. Other data generated during this study, including mBRRoK assay results, EC_50_ determinations, and cytotoxicity profiles, are available from the corresponding author upon reasonable request.

## Supplementary Materials

The following supporting information can be downloaded at https://www.mdpi.com/article/10.3390/biomedicines14030585/s1, Figure S1: Classification of lead compounds identified through the 6 h and 48 h mBRRoK assays. Data for lead compounds selected from 6 h mBRRoK assay (orange) and lead compounds selected from 48 h mBRRoK assay (blue) mapped onto the original 6 h (A) and 48 h (B) mBRRoK graphs to illustrate their positioning relative to one another and the benchmark antimalarial controls. The inset for (B) uses a cropped and expanded axis to better illustrate the lower left quadrant.; Figure S2: Concentration-dependent antiplasmodial activity of lead compounds from a library of microbial natural products. Compounds were selected as lead compounds from a larger library of microbial natural products showing promising antiplasmodial activity. A 3-fold dilution of compounds (3, 1, and 0.3 μg/mL) was incubated with synchronised trophozoites of Dd2^luc^ (A) and NF54^luc^ (B) parasite lines. Luciferase bioluminescence assay was carried after 48 h and % normalised growth established. Data plotted as mean % normalised growth (n = 6) ± standard deviation; Figure S3: Bioluminescence relative-rate-of-kill (BRRoK) plots of lead microbial natural products. The mean % normalised growth of P. falciparum Dd2^luc^ following 6 h (left column) and 48 h (right column) exposure to 9×, 3×, 1×, and 0.3× EC_50_ of compound (black) determined using luciferase bioluminescence assay. Data presented with chloroquine (red) and atovaquone (blue) controls (n = 9) ± SD); Figure S4: BRRoK plots of microbial natural products against Dd2^luc^ and NF54^luc^ *P. falciparum*. Compounds were incubated with 1–2% synchronised Dd2^luc^ (black) and NF54^luc^ (red) trophozoites. The mean % normalised growth compared to an untreated control was determined at 6 h (solid line) and 48 h (dotted line) using the luciferase bioluminescence assay. The mean % normalised growth (*n* = 9) was plotted ± SD against compound concentration (×EC_50_).

## Appendix A

Available data for the 36-lead microbial natural products provided by PhytoQuest upon request.

Available data for the 36-lead microbial natural products identified from the two-fixed concentration screen of Dd2^luc^ and NF54^luc^ to have inhibitory activity within 48 h. Some structural data are unknown (-).

**Compound**	**Molecular Weight**	**Molecular Formula**	**PhytoQuest Database Match**	**Organism Type**	**Genus**	**Specific Epithet**
**100050**	507.3349	C_23_H_45_NO_4_	Indaramycin analogue	Actinomyctes	*Streptomyces*	sp.
**100156**	523	-	-	Fungi	*Sarophorum*	*palmicola*
**100163**	523	-	-	Fungi	*Rosellinia*	*helvetica*
**100166**	491	-	-	Fungi	*Rosellinia*	*helvetica*
**100167**	228.0787	-	5-chloro-4, 6-dimethoxyphthalide	Fungi	*Tulostoma*	*brumale*
**100180**	530	-	-	Fungi	*Myrothecium*	*roridum*
**100181**	642	-	Trichoverritone	Fungi	*Myrothecium*	*roridum*
**100260**	562	-	-	Actinomycetes	*Streptomyces*	sp.
**100267**	263	-	-	Fungi	*Chaunopycnis*	*alba*
**100288**	263.1521	C_15_H_21_NO_3_	Pyridoxatin	Fungi	*Hirsutella*	sp.
**100407**	308.1263	C_16_H_20_O_6_	-	Fungi	*Exserohilum*	*protrudens*
**100476**	548.31	-	-	Actinomycetes	*Streptomyces*	sp.
**100498**	239.0946	C_15_H_13_NO_2_ tentative	-	Fungi	*Aspergillus*	*ustus*
**100567**	530.1242	-	-	Fungi	*Nectria*	*flavoviridis*
**100568**	532.1408	C_29_H_24_O_10_	Chaetochromin C	Fungi	*Nectria*	*flavoviridis*
**100569**	546.1548	C_30_H_26_O_10_	Chaetochromin A	Fungi	*Nectria*	*flavoviridis*
**100570**	544.1401	-	-	Fungi	*Nectria*	*flavoviridis*
**100576**	518.1226	C_28_H_22_O_10_	Cephalochromin	Fungi	*Nectria*	*flavoviridis*
**100585**	530.2523	C_29_H_38_O_9_	Roridin D	Fungi	*Myrothecium*	*verrucaria*
**100596**	428.2567	C_26_H_36_O_5_	-	Fungi	*Fusicoccum*	*cf aesculi*
**100648**	359.2084	C_21_H_29_NO_4_	Plant alkaloid	Fungi	*Chaetosphaeria*	*myriocarpa*
**100657**	292.1317	C_16_H_20_O_5_	Asperentin	Fungi	*Eurotium*	*herbariorum*
**100669**	614.2753	C_33_H_42_O_11_	-	Fungi	*Myrothecium*	*verrucaria*
**100670**	512.2388	C_29_H_36_O_8_	Roridin H	Fungi	*Myrothecium*	*verrucaria*
**100686**	662.2175	C_32_H_38_O_15_	Antibiotic Bk223B or NG011	Fungi	*Penicillium*	*cf aculeatum*
**100705**	292.1674	C_17_H_24_O_4_	Trichodermin	Fungi	*Trichoderma*	sp.
**100735**	429.2885	C_26_H_39_NO_4_	Tetramic acid derivative PF1052/antibiotic AB 4063B 1,2-epoxide	Fungi	*Coniothyrium*	*fuckelii*
**101158**	507.125	-	-	Fungi	*Xylaria*	sp.
**101159**	491.2664	C_30_H_37_NO_5_	Zygosporin G	Fungi	*Xylaria*	sp.
**101160**	508.1332	-	-	Fungi	*Xylaria*	sp.
**101161**	507.2621	C_30_H_37_NO_6_ tentative	Isomeric with Cytochalasin C and D	Fungi	*Xylaria*	sp.
**101173**	516.10069	C_29_H_16_N_4_O_6_	-	Fungi	*Nectria*	*vilior*
**101271**	491.2704	C_30_H_37_NO_5_ tentative	-	Fungi	*Xylaria*	sp.
**101326**	358.2142	C_22_H_30_O_4_	-	Fungi	*Macrophomina*	*phaseolina*
**101367**	263	C_15_H_21_NO_3_	-	Fungi	*Chaunopycnis*	*alba*
**101371**	466	-	-	Fungi	*Chaetomium*	*convolutum*

## References

[1] Perez-Moreno G., Cantizani J., Sanchez-Carrasco P., Ruiz-Perez L.M., Martin J., El Aouad N., Perez-Victoria I., Tormo J.R., Gonzalez-Menendez V., Gonzalez I. (2016). Discovery of New Compounds Active against Plasmodium falciparum by High Throughput Screening of Microbial Natural Products. PLoS ONE.

[2] Moyo P., Mugumbate G., Eloff J.N., Louw A.I., Maharaj V.J., Birkholtz L.M. (2020). Natural Products: A Potential Source of Malaria Transmission Blocking Drugs?. Pharmaceuticals.

[3] Yoo E., Schulze C.J., Stokes B.H., Onguka O., Yeo T., Mok S., Gnadig N.F., Zhou Y., Kurita K., Foe I.T. (2020). The Antimalarial Natural Product Salinipostin A Identifies Essential alpha/beta Serine Hydrolases Involved in Lipid Metabolism in *P. falciparum* Parasites. Cell Chem. Biol..

[4] Tan K.R., Magill A.J., Parise M.E., Arguin P.M. (2011). Doxycycline for malaria chemoprophylaxis and treatment: Report from the CDC expert meeting on malaria chemoprophylaxis. Am. J. Trop. Med. Hyg..

[5] Berdy J. (1974). Recent developments of antibiotic research and classification of antibiotics according to chemical structure. Adv. Appl. Microbiol..

[6] Hameed H., King E.F.B., Doleckova K., Bartholomew B., Hollinshead J., Mbye H., Ullah I., Walker K., Van Veelen M., Abou-Akkada S.S. (2021). Temperate Zone Plant Natural Products-A Novel Resource for Activity against Tropical Parasitic Diseases. Pharmaceuticals.

[7] Forte B., Ottilie S., Plater A., Campo B., Dechering K.J., Gamo F.J., Goldberg D.E., Istvan E.S., Lee M., Lukens A.K. (2021). Prioritization of Molecular Targets for Antimalarial Drug Discovery. ACS Infect. Dis..

[8] Famodimu M.T. (2020). Development and Validation of A Bioluminescence Assay for High Throughput Screening of Potent and Rapidly Cytocidal Compounds Against Intraerythrocytic *Plasmodium falciparum*. Ph.D. Thesis.

[9] Ullah I., Sharma R., Biagini G.A., Horrocks P. (2017). A validated bioluminescence-based assay for the rapid determination of the initial rate of kill for discovery antimalarials. J. Antimicrob. Chemother..

[10] Alven S., Aderibigbe B. (2019). Combination Therapy Strategies for the Treatment of Malaria. Molecules.

[11] Ullah I., Sharma R., Mete A., Biagini G.A., Wetzel D.M., Horrocks P.D. (2020). The relative rate of kill of the MMV Malaria Box compounds provides links to the mode of antimalarial action and highlights scaffolds of medicinal chemistry interest. J. Antimicrob. Chemother..

[12] Trager W., Jensen J.B. (1976). Human malaria parasites in continuous culture. Science.

[13] Wong E.H., Hasenkamp S., Horrocks P. (2011). Analysis of the molecular mechanisms governing the stage-specific expression of a prototypical housekeeping gene during intraerythrocytic development of *P. falciparum*. J. Mol. Biol..

[14] Hmoud M.K. (2019). Exploring the Erythrocyte Invasion-Blocking Effect of Modified Heparin and Heparin Mimetics in the Human Malaria Parasite *Plasmodium falciparum*. Ph.D. Thesis.

[15] Hasenkamp S., Sidaway A., Devine O., Roye R., Horrocks P. (2013). Evaluation of bioluminescence-based assays of anti-malarial drug activity. Malar. J..

[16] Sanz L.M., Crespo B., De-Cozar C., Ding X.C., Llergo J.L., Burrows J.N., Garcia-Bustos J.F., Gamo F.J.P. (2012). falciparum in vitro killing rates allow to discriminate between different antimalarial mode-of-action. PLoS ONE.

[17] Asano D., Takakusa H., Nakai D. (2023). Oral Absorption of Middle-to-Large Molecules and Its Improvement, with a Focus on New Modality Drugs. Pharmaceutics.

[18] Isaka M., Jaturapat A., Kladwang W., Punya J., Lertwerawat Y., Tanticharoen M., Thebtaranonth Y. (2000). Antiplasmodial compounds from the wood-decayed fungus *Xylaria* sp. BCC 1067. Planta Medica.

[19] Isaka M., Tanticharoen M., Kongsaeree P., Thebtaranonth Y. (2001). Structures of cordypyridones A-D, antimalarial N-hydroxy- and N-methoxy-2-pyridones from the insect pathogenic fungus Cordyceps nipponica. J. Org. Chem..

[20] Saint-Leger A., Sinadinos C., Ribas de Pouplana L. (2016). The growing pipeline of natural aminoacyl-tRNA synthetase inhibitors for malaria treatment. Bioengineered.

[21] Liu X., Li R.Q., Zeng Q.X., Li Y.Q., Chen X.A. (2023). A Novel Zn_2_Cys_6_ Transcription Factor, TopC, Positively Regulates Trichodin A and Asperpyridone A Biosynthesis in Tolypocladium ophioglossoides. Microorganisms.

[22] Lakornwong W., Kanokmedhakul K., Soytong K., Unartngam A., Tontapha S., Amornkitbamrung V., Kanokmedhakul S. (2019). Types A and D Trichothecene Mycotoxins from the Fungus Myrothecium roridum. Planta Medica.

[23] Hoepfner D., McNamara C.W., Lim C.S., Studer C., Riedl R., Aust T., McCormack S.L., Plouffe D.M., Meister S., Schuierer S. (2012). Selective and specific inhibition of the plasmodium falciparum lysyl-tRNA synthetase by the fungal secondary metabolite cladosporin. Cell Host Microbe.

[24] Isaka M., Punya J., Lertwerawat Y., Tanticharoen M., Thebtaranonth Y. (1999). Antimalarial activity of macrocyclic trichothecenes isolated from the fungus Myrothecium verrucaria. J. Nat. Prod..

[25] Ghorbal M., Gorman M., Macpherson C.R., Martins R.M., Scherf A., Lopez-Rubio J.J. (2014). Genome editing in the human malaria parasite Plasmodium falciparum using the CRISPR-Cas9 system. Nat. Biotechnol..

[26] Wagner J.C., Platt R.J., Goldfless S.J., Zhang F., Niles J.C. (2014). Efficient CRISPR-Cas9-mediated genome editing in Plasmodium falciparum. Nat. Methods.

