# Supplementary material for: Novel Antiplasmodial Natural Products Identified Through a Modified Bioluminescence-Based Rate-of-Kill Assay
Source: Biomedicines. 2026 Mar 5;14(3):585. doi: 10.3390/biomedicines14030585 (PMC13024254; doi:10.3390/biomedicines14030585)
Supplement: Supplementary file 1 [file biomedicines-14-00585-s001.zip › biomedicines-4099121-supplementary.pdf]

Supplementary materials

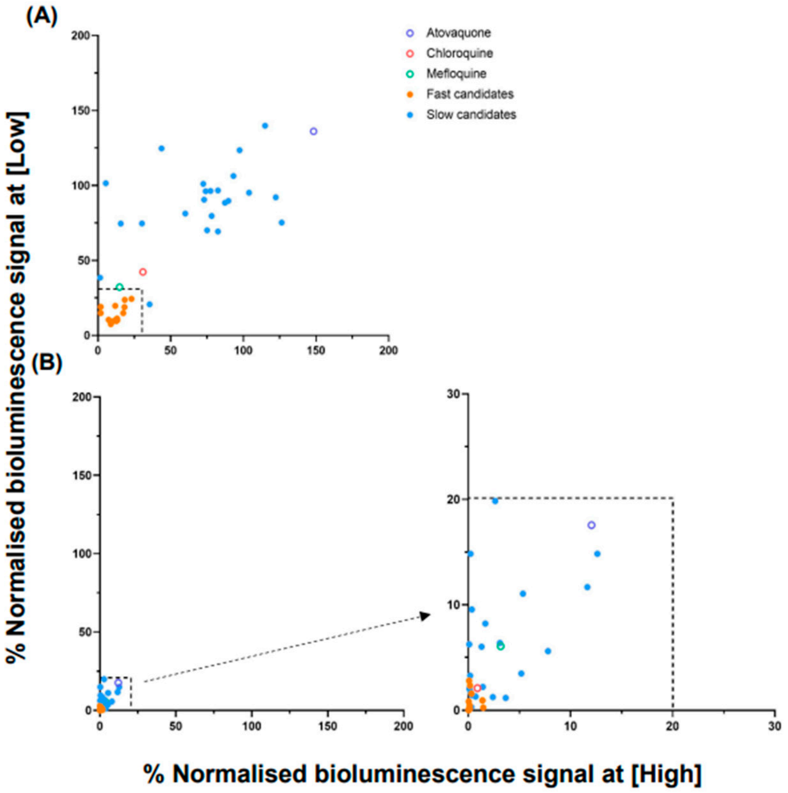

Supplementary Figure S1. Classification of lead compounds identified through the 6hr and 48hr mBRRoK assays. *Data for lead compounds selected from 6hr mBRRoK assay (orange) and lead compounds selected from 48-hour mBRRoK assay (blue) mapped onto the original 6-hour (A) and 48-hour (B) mBRRoK graphs to illustrate their positioning relative to one another and the benchmark antimalarial controls. The inset for (B) uses a cropped and expanded axis to better illustrate the lower left quadrant.*

(A)

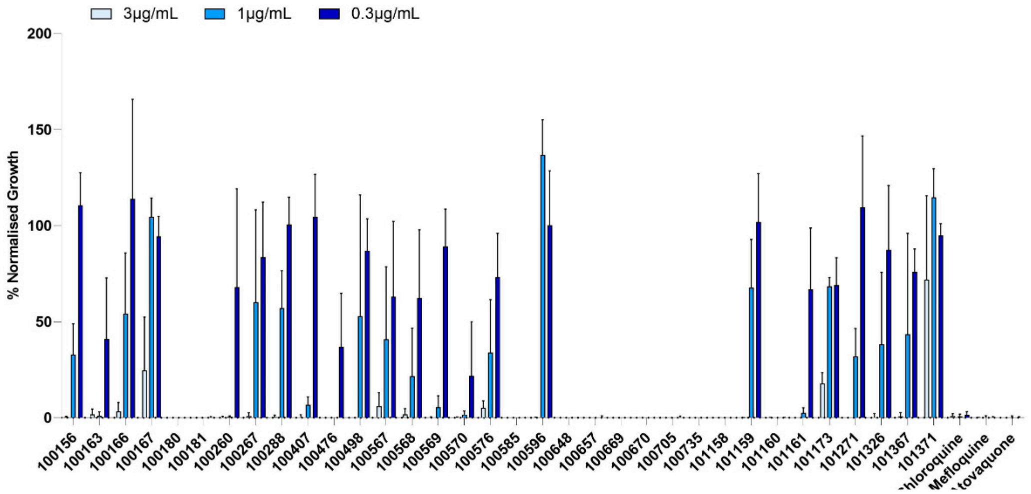

(B)

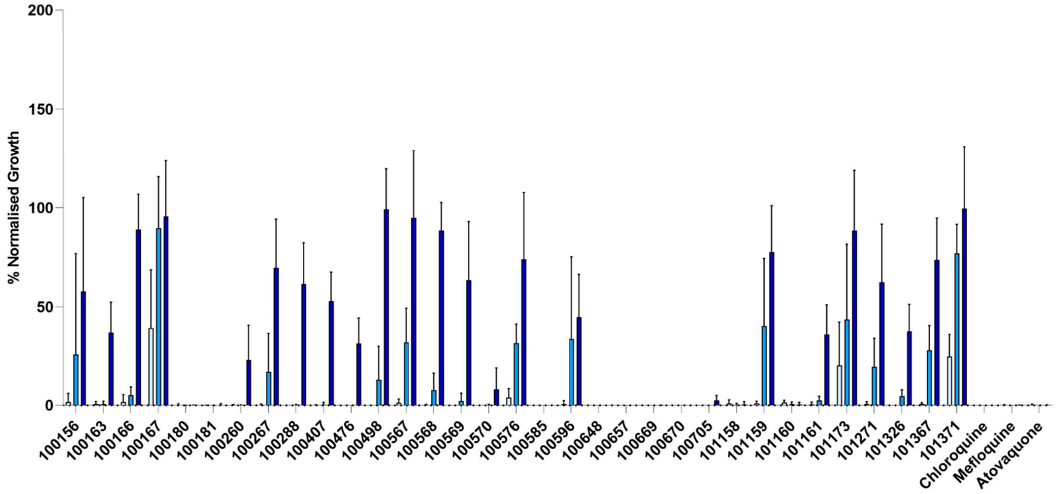

Supplementary Figure S2. Concentration-dependent antiplasmodial activity of lead compounds from a library of microbial natural products. *Compounds were selected as lead compounds from a larger library of microbial natural products showing promising antiplasmodial activity. A 3-fold dilution of compounds (3, 1 and 0.3µg/mL) was incubated with synchronised trophozoites of Dd2^{luc}* ***(A)*** *and NF54^{luc}* ***(B)*** *parasite lines. Luciferase bioluminescence assay was carried after 48 hours and % normalised growth established. Data plotted as mean % normalised growth (n=6) ±standard deviation.*

6 hours **48 hours**

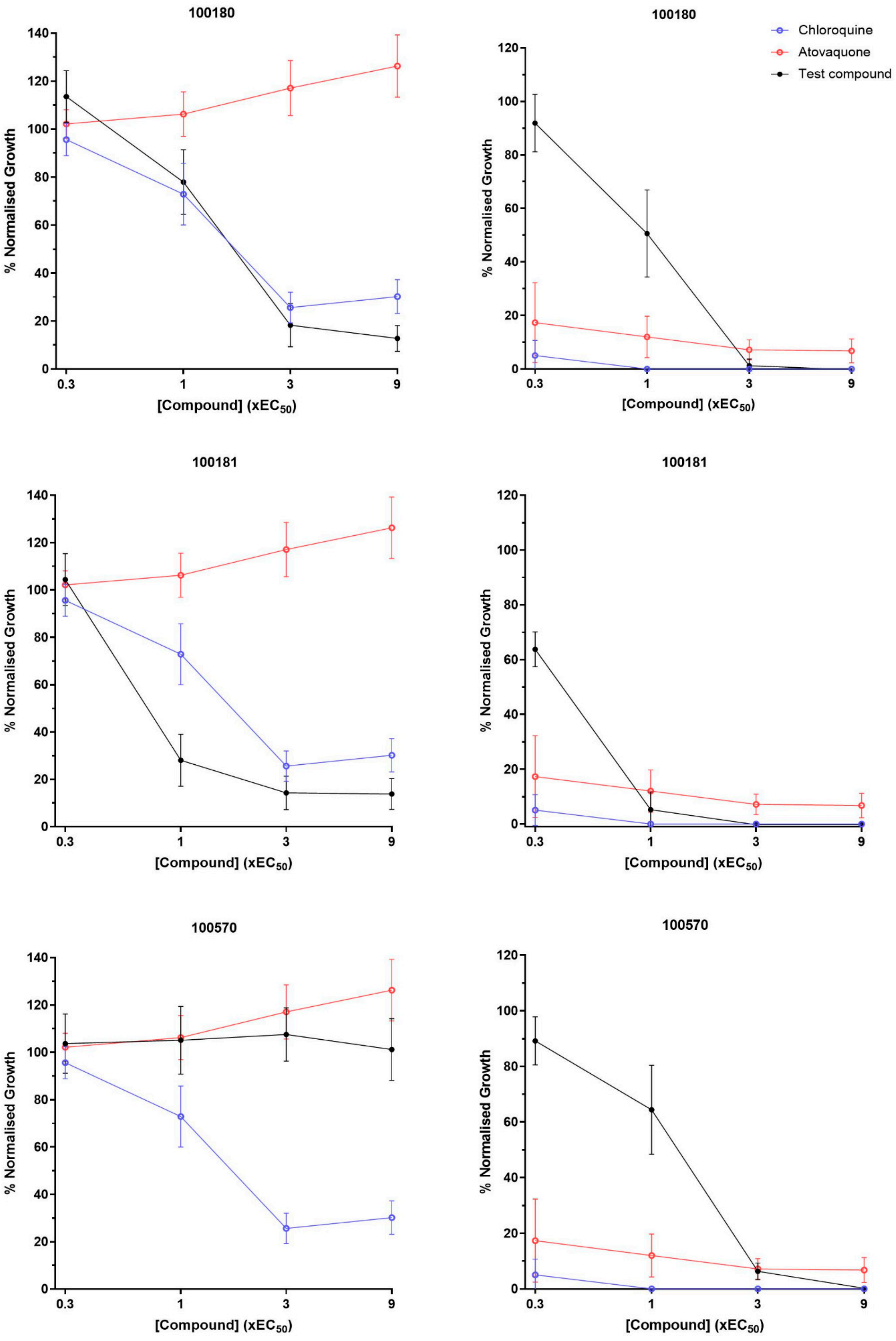

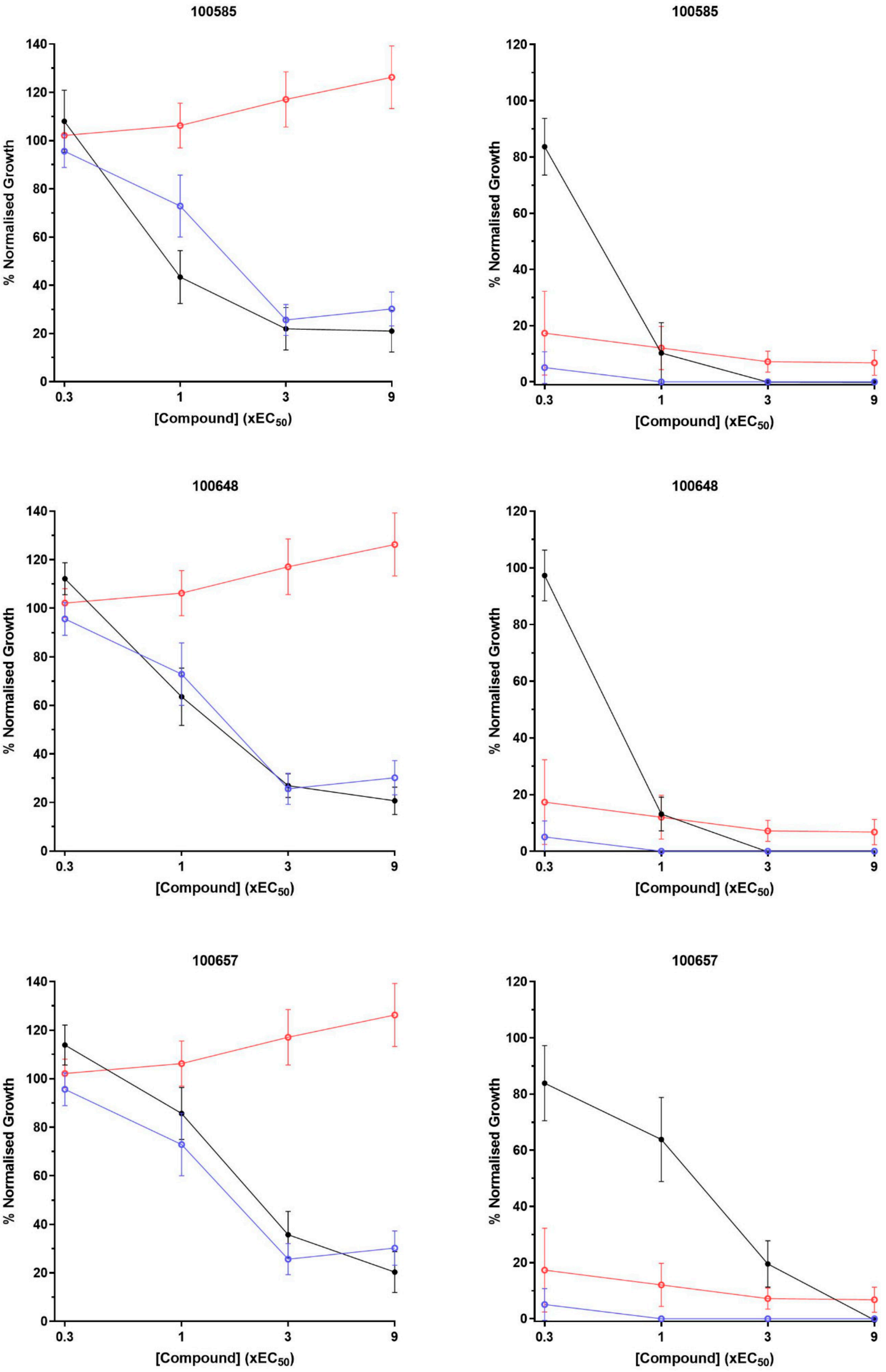
100585
100585
100648
100648
100657
100657
% Normalised Growth
[Compound] (xEC50)
0.3
1
3
9
0
20
40
60
80
100
120
140

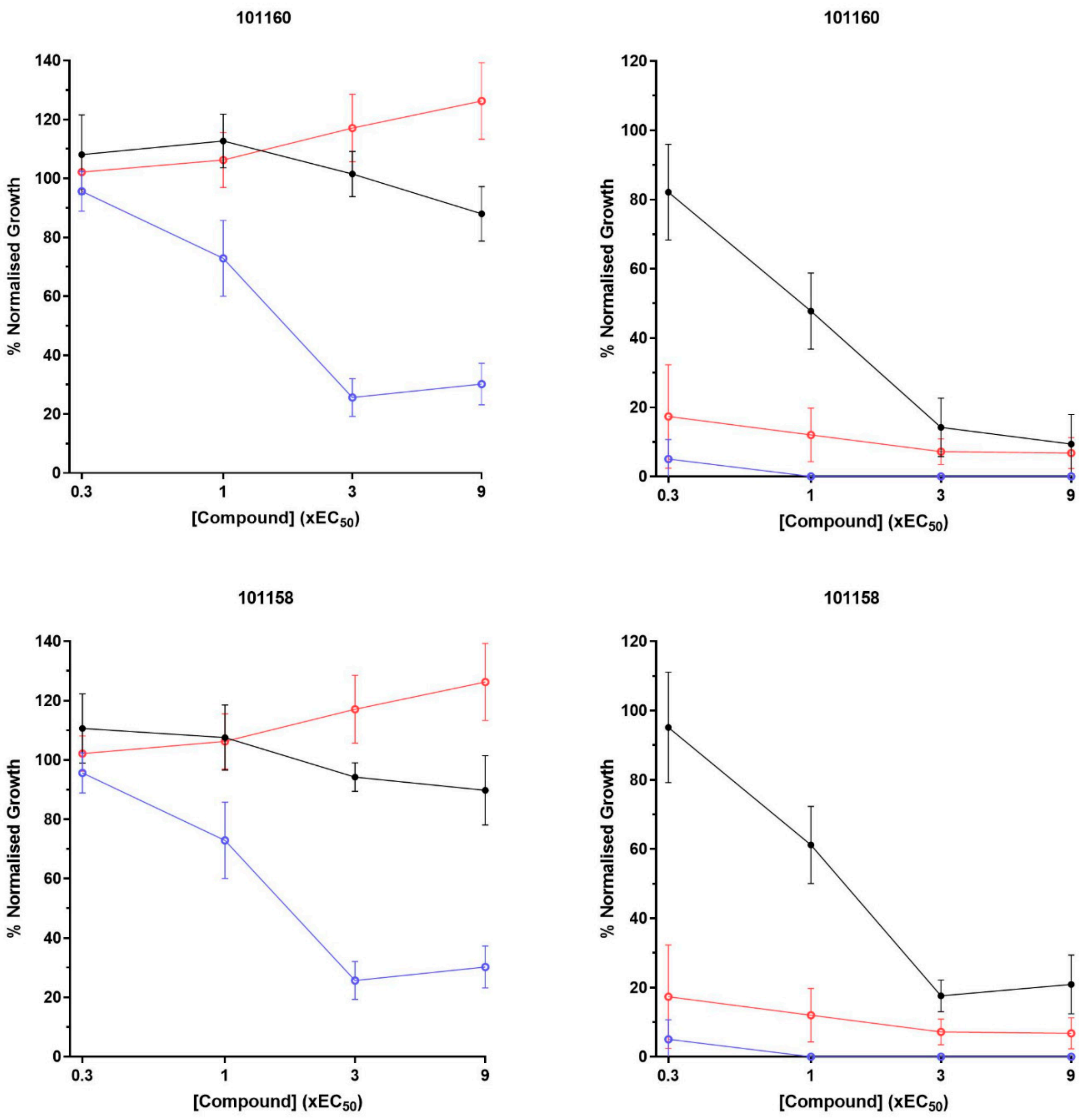
101160
% Normalised Growth
[Compound] (xEC50)
101160
% Normalised Growth
[Compound] (xEC50)
101158
% Normalised Growth
[Compound] (xEC50)
101158
% Normalised Growth
[Compound] (xEC50)

Supplementary Figure S3. Bioluminescence Relative Rate of Kill (BRRoK) plots of lead microbial natural products. *The mean % normalized growth of P. falciparum Dd2*^{luc} *following 6-hour (left column) and 48-hour (right column) exposure to 9x, 3x, 1x and 0.3xEC*$_{50}$ *of compound (black) determined using luciferase bioluminescence assay. Data presented with chloroquine (red) and atovaquone (blue) controls (n=9 ±SD).*

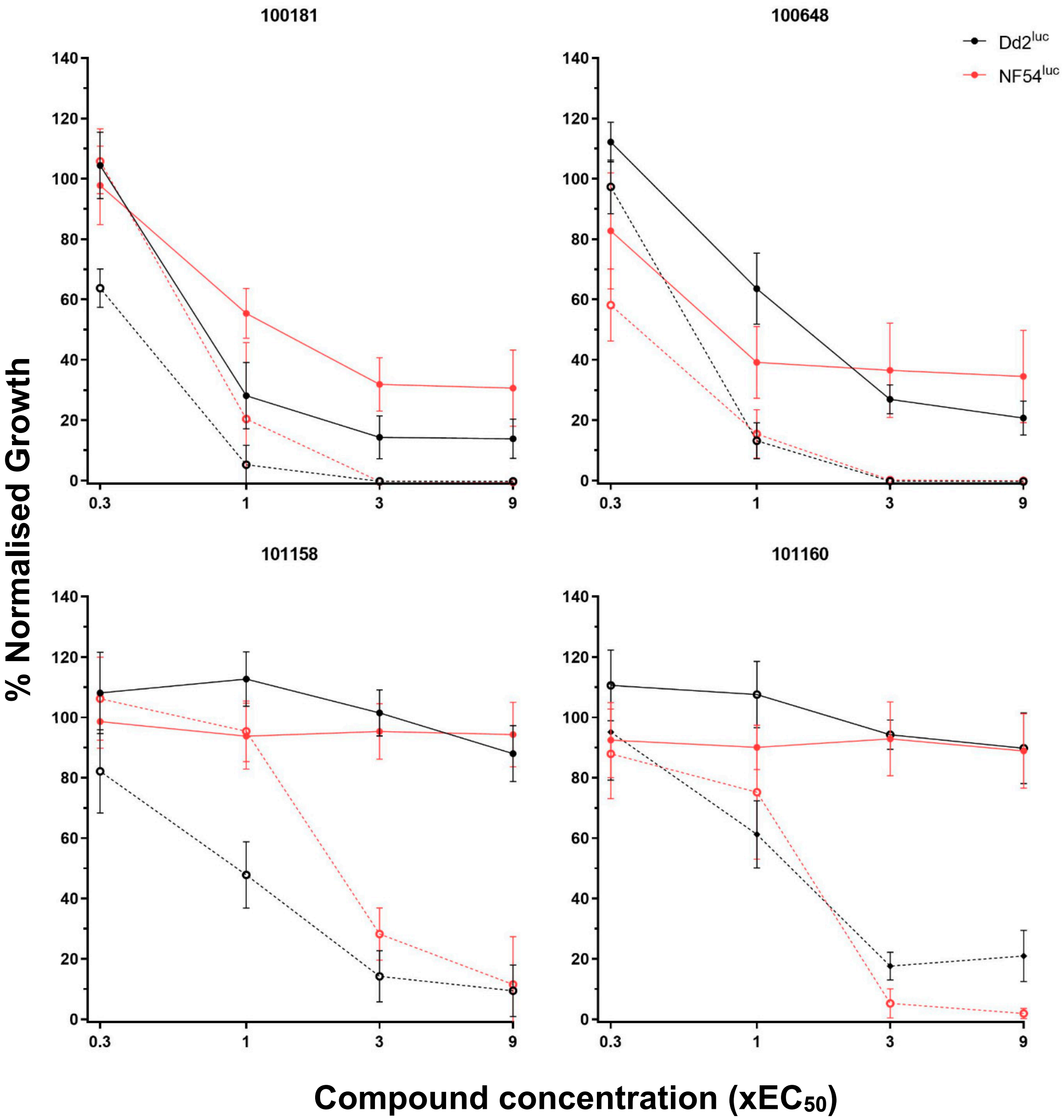

Supplementary Figure S4. BRRoK plots of microbial natural products against Dd2luc and NF54luc P. falciparum. *Compounds were incubated with 1-2% synchronized Dd2*^{luc} *(black) and NF54*^{luc} *(red) trophozoites. The mean % normalized growth compared to an untreated control was determined at 6 hours (solid line) and 48 hours (dotted line) using the luciferase bioluminescence assay. The mean % normalized growth (n=9) was plotted ± SD against compound concentration (xEC*$_{50}$*).*
